# Changes in Thoracic Cavity Volume After Bilateral Lung Transplantation

**DOI:** 10.3389/fmed.2022.881119

**Published:** 2022-05-26

**Authors:** Woo Sik Yu, Chul Hwan Park, Hyo Chae Paik, Jin Gu Lee, Seulgi You, Jaeyong Shin, Junho Jung, Seokjin Haam

**Affiliations:** ^1^Department of Thoracic and Cardiovascular Surgery, Ajou University School of Medicine, Suwon, South Korea; ^2^Department of Radiology and the Research Institute of Radiological Science, Yonsei University Health System, Seoul, South Korea; ^3^Department of Thoracic and Cardiovascular Surgery, Severance Hospital, Yonsei University College of Medicine, Seoul, South Korea; ^4^Department of Radiology, Ajou University School of Medicine, Suwon, South Korea; ^5^Department of Preventive Medicine, Yonsei University College of Medicine, Seoul, South Korea

**Keywords:** lung transplantation, thoracic cavity volume, restrictive lung disease, obstructive lung disease, chest wall remodeling

## Abstract

**Purpose:**

End-stage lung diseases result in anatomical changes of the thoracic cavity. However, very few studies have assessed changes in the thoracic cavity after lung transplantation (LTx). This study aimed to evaluate the relationships between thoracic cavity volume (TCV) changes after LTx and underlying lung disease.

**Methods:**

We reviewed 89 patients who underwent a pre-LTx pulmonary function test (PFT), chest computed tomography (CT) scan, and 1-year follow-up CT after LTx. These patients were classified into two groups according to pre-LTx PFT as follows: obstructive group [forced expiratory volume in 1 s (FEV1)/forced vital capacity (FVC) ratio < 70%] and restrictive group (FEV1/FVC ratio > 70%). We measured TCV using CT scan before and at 1 year after LTx and compared the TCV change in the two groups.

**Results:**

In the restrictive group, TCV increased after LTx (preop: 2,347.8 ± 709.5 mL, 1-year postop: 3,224.4 ± 919.0 mL, *p* < 0.001). In contrast, in the obstructive group, it decreased after LTx (preop: 4,662.9 ± 1,296.3 mL, 1-year postop: 3,711.1 ± 891.7 mL, *p* < 0.001). We observed that restrictive lung disease, taller stature, lower body mass index, and larger donor lung were independently associated with increased TCV after LTx.

**Conclusion:**

The disease-specific chest remodeling caused by restriction and hyperinflation is at least, in part, reversible. After LTx, the chest remodeling appears to occur in the opposite direction to the disease-specific remodeling caused by the underlying lung disease in recipients.

## Introduction

Significant lung size mismatch between the donor and the recipient may cause serious adverse effects during and after lung transplantation (LTx). An oversized graft can lead to inability to close the chest, impaired hemodynamics, persistent atelectasis, and airway complications (1). In contrast, an undersized graft can cause residual space problems, such as persistent pneumothorax and pleural effusions (2, 3). Therefore, lung size matching is an essential component in LTx, and lung transplant surgeons try to achieve it. Various size matching criteria have been proposed to avoid significant size mismatching. Predicted total lung capacity (pTLC), which is calculated using sex and height, is currently the most commonly used criterion (4, 5). Sometimes, transplant surgeons reject organs that are believed to be too large or too small for the patients and resect donor lung parenchyma to reduce the graft volumes (3, 6).

Depending on the type of end-stage lung disease, the patients’ chest wall can change to some extent, and this is referred to as disease-specific chest remodeling (3). Patients with restrictive lung diseases, such as idiopathic pulmonary fibrosis (IPF), typically have decreased lung volume and a smaller thoracic cavity, while patients with obstructive lung diseases, such as chronic obstructive pulmonary disease (COPD), typically have increased lung volume and a larger thoracic cavity, compared to the normal population (7–10). Based on these facts, we assumed that the thoracic cavity could change after LTx either.

In this study, we measured the changes in thoracic cavity volume (TCV) before and after bilateral LTx using chest computed tomography (CT) scans and investigated the factors that were associated with these changes. Such postoperative changes should be considered by surgeons when selecting an appropriate lung graft size.

## Materials and Methods

### Patients

From January 2007 to September 2017, 212 patients underwent lung transplantation (LTx) at the Yonsei University College of Medicine or Ajou University School of Medicine. Patients were excluded from the study if they met the following criteria: (1) underwent single lung transplantation or combined solid organ transplantation (*n* = 17); (2) did not have preoperative PFT (PFT) data (*n* = 34); (3) did not undergo 1-year postoperative chest CT scan (*n* = 69); and (4) had a significant abnormality, such as pleural effusion (*n* = 2) or consolidation (*n* = 2) on 1-year chest CT.

**FIGURE 1:**
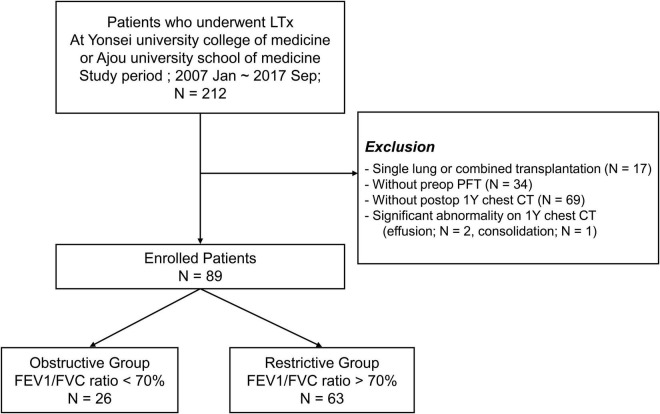
Study population selection. LTx, lung transplantation; preop, pre-operative; postop, postoperative; 1Y, 1 year.

In total, 89 patients who met the aforementioned criteria were reviewed and classified into the obstructive (*n* = 26) and restrictive (*n* = 63) groups according to the GOLD criteria based on PFT results (11) (Figure 1). This retrospective study was approved by the Institutional Review Board of each hospital (Severance Hospital: 4-2020-0671 and Ajou University Hospital: AJIRB-MED-MDB-20-245), and the need for informed consent was waived.

### Size Matching and Lung Transplantation Procedure

pTLC has been used for the donor and recipient size matching at the two institutions, and the equations proposed by Goldman and Becklake were used to calculate pTLC as follows (12):


pTLC⁢for⁢men=[-0.015×age]+[0.094×height⁢in⁢cm]-9.17

pTLC⁢for⁢women=[-0.008×age]+[0.079×height⁢in⁢cm]-7.49

pTLC ratio (donor pTLC/recipient pTLC) between 0.75 and 1.25 was considered acceptable. However, the donor lung graft was used at the discretion of the attending surgeon considering various factors such as patients’ urgency.

The previously described (13) surgical procedure was nearly identical at the two institutions. Briefly, all lung grafts were recovered en bloc from brain-dead donors. In recipients, a clamshell incision in the fourth intercostal space is the preferred method of surgical incision. Bilateral LTx was performed sequentially. If necessary, graft volume reduction, including wedge resections or lobectomies, was performed due to the excess size of the donor’s lung compared to the size of the recipient’s chest wall. The decision to perform graft volume reduction was made by the attending surgeon.

All organs used for transplantation in this study were provided by the government agency, the Korean Network for Organ Sharing (KONOS). The entire process for transplantation was strictly regulated by the relevant legislation. None of the transplant donors was from a vulnerable population, and all donors or next of kin provided written informed consent that was freely given.

### Thoracic Cavity Volume Measurement

All patients underwent chest CT scan before and in the first year after LTx. CT was performed from the lung apices to the adrenal glands during breath holding at the end of inspiration with the subject in the supine position. After acquiring the scout image to determine the field of view, a CT scan was performed using a helical technique at a 1-mm reconstruction interval using the mediastinal window setting. The exposure parameters for the CT scans were as follows: 120 kVp, 50–130 mA, and 1–3-mm slice thickness. The conventional CT images were reconstructed using 1–3-mm reconstruction increment on the scanner workstation. Two radiologists (CHP and SY) reviewed all CT images. Eligible axial CT images were transferred to a commercially available reconstruction program using three-dimensional (3D) reconstruction and measurement (Aquarius iNtuition™ Ver. 4.4.6; TeraRecon, Foster City, CA, United States). TCV was defined as the anatomical volume of both lungs and measured using a threshold-based three-dimensional segmentation technique. First, both lungs were selected and extracted automatically using an automatic segmentation technique. The default threshold was set to −200 to −1,024 HU. Second, the included large airways (trachea and bilateral main bronchi) were carefully selected manually and excluded. Then the extracted three-dimensional lungs were visually confirmed. Finally, the anatomical lung volume was measured and used as TCV (Supplementary Figure 1). ΔTCV was defined as the TCV at the first year after LTx minus the preoperative TCV.

### Donor/Recipient Size Discrepancy

#### Donor Predicted Total Lung Capacity/Recipient Predicted Total Lung Capacity

Because donor lung volume and recipient thoracic cavity volume before the development of pulmonary disease cannot be measured directly, we used donor pTLC/recipient pTLC as a surrogate for donor lung size compared to the non-diseased recipient’s thoracic cavity volume.

#### Donor Predicted Total Lung Capacity/Recipient Thoracic Cavity Volume

Preoperative recipient TCV (TCV_preop_) represents thoracic cavity volume of the recipient with end-stage lung disease. We used donor pTLC/recipient TCV_preop_ as a surrogate for the lung size of donors compared to the diseased recipient’s thoracic cavity volume.

### Statistical Analysis

Statistical analyses were performed using SPSS version 25.0 (IBM Corp., Armonk, NY, United States) and R version 3.6.1 (R Foundation for Statistical Computing, Vienna, Austria) software. Continuous variables were compared using Student’s *t*-test or the Mann–Whitney *U* test. Categorical variables were compared using the chi-square test or Fisher’s exact test. A paired-sample *t*-test was implemented for within-group comparisons between preoperative and 1-year postoperative lung volumes. Analysis of covariance (ANCOVA) was used to compare the groups in terms of preoperative and 1-year postoperative TCV after adjusting for recipient pTLC. The correlations between variables were calculated using the Pearson correlation test. Multivariate logistic regression analysis was performed to identify independent factors associated with ΔTCV. Clinically relevant variables for TCV were included in the model (age, sex, height, body mass index (BMI), donor pTLC/recipient TCV, and lung volume reduction). A *p*-value of < 0.05 was considered statistically significant.

## Results

### Patient Characteristics

Patient characteristics are summarized in Table 1. The mean age of the 89 participants was 48.7 ± 12.1 years. Forty-eight patients (53.9%) were male. The most common indication for LTx was IPF, which 44 patients (49.4%) had. The restrictive group comprised 63 patients (70.3%), and these patients tended to be older and had a higher BMI than those in the obstructive group.

**TABLE 1:** Baseline characteristics of the patients.

	Total (*n* = 89)	Restrictive (*n* = 63)	Obstructive (*n* = 26)	*P*-value
Age, years	48.7 ± 12.2	51.1 ± 10.3	42.9 ± 14.5	0.012
Male	48 (53.9%)	38 (60.3%)	10 (38.5%)	0.100
Height, cm	164.1 ± 7.0	164.4 ± 7.4	163.4 ± 6.2	0.563
BMI, kg/m^2^	20.2 ± 4.0	21.1 ± 3.7	18.0 ± 3.8	0.001
Diagnosis, *n* (%)				< 0.001
Bronchiectasis	8 (9.0%)	2 (3.2%)	5 (19.2%)	
COPD	3 (3.4%)	0 (0%)	3 (11.5%)	
CTD-ILD	10 (11.2%)	10 (15.9%)	0 (0.0%)	
GVHD	11 (12.4%)	4 (6.3%)	7 (26.9%)	
IPF	44 (49.4%)	43 (68.3%)	1 (3.8%)	
LAM	7 (7.9%)	0 (0.0%)	7 (26.9%)	
Other—ILD	6 (6.7%)	3 (4.8%)	3 (11.5%)	
FEV1% predicted	38.8 ± 17.3	44.5 ± 15.7	25.0 ± 13.1	< 0.001
FVC% predicted	40.4 ± 14.0	37.4 ± 12.0	47.8 ± 15.8	0.001
FEV1/FVC (%)	73.5 ± 25.6	88.6 ± 7.7	36.8 ± 13.8	< 0.001
Lung volume reduction	30 (33.7%)	22 (34.9%)	8 (30.8%)	0.896
Procedure for lung volume reduction				0.380
Lower lobectomy	2 (2.2%)	2 (3.2%)	0 (0.0%)	
Middle lobectomy	1 (1.1%)	0 (0.0%)	1 (3.8%)	
Middle lobectomy + Wedge	2 (2.2%)	2 (3.2%)	0 (0.0)	
Wedge, multiple	15 (16.9%)	12 (19.0%)	3 (11.5%)	
Wedge, single	10 (11.2%)	6 (9.5%)	4 (15.4%)	
Donor/recipient size discrepancy				
Recipient pTLC, mL	5,342.5 ± 774.1	5,377.2 ± 808.3	5,258.2 ± 691.8	0.513
Donor pTLC, mL	5,613.7 ± 839.9	5,533.4 ± 799.0	5,808.4 ± 918.6	0.161
Donor pTLC/recipient pTLC,%	106.2 ± 16.0	104.2 ± 16.0	111.0 ± 15.2	0.071
Donor pTLC/recipient TCV_preop_,%	221.5 ± 97.2	257.9 ± 90.1	133.3 ± 41.5	< 0.001
Recipient TCV				
Preop TCV, mL	3,024.1 ± 1,397.5	2,347.8 ± 709.5	4,662.9 ± 1,296.3	< 0.001
1-Year TCV, mL	3,366.6 ± 933.0	3,224.4 ± 919.0	3,711.1 ± 891.7	0.024
ΔTCV, Ml	342.5 ± 1,134.3	876.6 ± 669.6	−951.8 ± 977.6	< 0.001
1-Year PFT ^a^				
FVC% predicted	66.0 ± 18.6	64.9 ± 19.7	69.2 ± 14.7	0.377
FEV1% predicted	73.3 ± 20.9	72.2 ± 21.8	76.5 ± 17.8	0.431
FEV1/FVC (%)	84.9 ± 11.1	84.1 ± 11.1	87.2 ± 11.1	0.277

*BMI, body mass index; COPD, chronic obstructive pulmonary disorder; CTD-ILD, connective tissue disease-interstitial lung disease; GVHD, graft-versus-host disease; IPF, idiopathic pulmonary fibrosis; LAM, lymphangioleiomyomatosis; ILD, interstitial lung disease; FEV1, forced expiratory volume in 1 s; FVC, forced vital capacity; pTLC, predicted total lung capacity; TCV, thoracic cavity volume.*

*(ΔTCV = 1-year postoperative TCV – preoperative TCV), PFT, pulmonary function test; FVC, forced vital capacity; FEV1, forced expiratory volume in 1 s.*

*^a^Postoperative 1-year pulmonary function test data were available in 78 patients.*

### Predicted Total Lung Capacity and Thoracic Cavity Volume of Donor/Recipient

The donor/recipient pTLC of all patients was 106.2% ± 16.0%. The donor/recipient pTLC was not significantly different between the two groups (restrictive group [104.2% ± 16.0%] vs. obstructive group [111.0% ± 15.2%], *p* = 0.071). However, the restrictive group had a significantly higher donor pTLC/recipient TCV than the obstructive group [restrictive group (257.9% ± 90.1%) vs. obstructive group (133.3% ± 41.5%), *p* < 0.001].

### Thoracic Cavity Volume Changes Before and After Lung Transplantation

**FIGURE 2:**
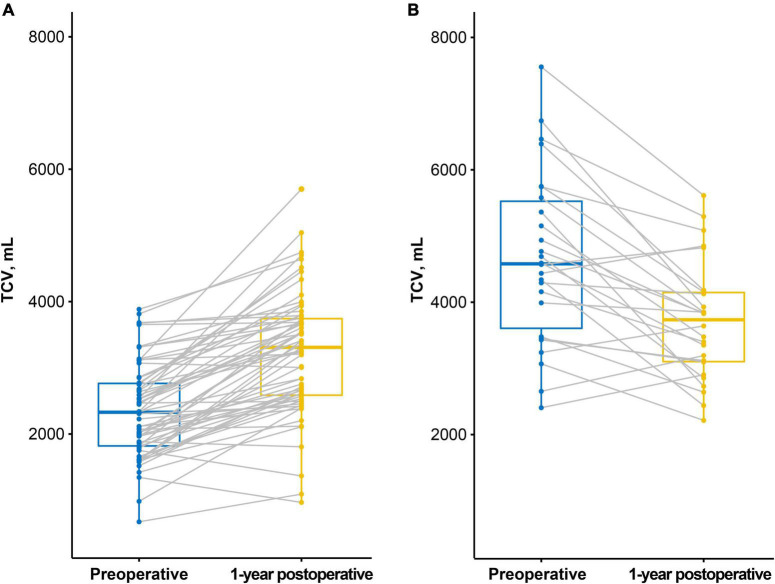
Preoperative and 1-year postoperative thoracic cavity volume (TCV) measured by CT. **(A)** In the restrictive group, TCV increased after lung transplantation (LTx). **(B)** In the obstructive group, TCV decreased after LTx.

In the restrictive group, TCV increased after LTx (preop: 2,347.8 ± 709.5 mL, 1-year postop: 3,224.4 ± 919.0 mL, *p* < 0.001). In contrast, in the obstructive group, it decreased after LTx (preop: 4,662.9 ± 1,296.3 mL, 1-year postop: 3,711.1 ± 891.7 mL, *p* < 0.001, Figure 2).

**FIGURE 3:**
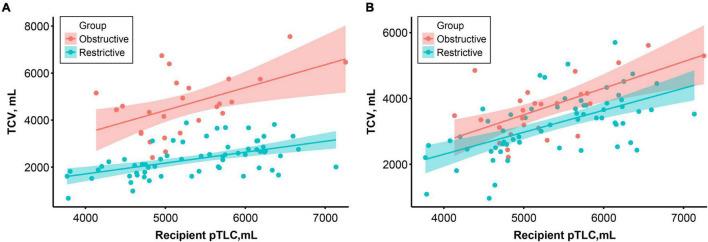
Correlation between recipient predictive total lung capacity (pTLC) and preoperative **(A)** and 1-year postoperative **(B)** thoracic cavity volume according to the study groups. Colored lines and areas indicate the regression lines and 95% confidence intervals, respectively.

ANCOVAs were performed to compare the TCV in the two groups adjusting for pTLC. There was a significant difference in preoperative TCV between the groups (*F* = 89.81, *p* < 0.001). After transplantation, the difference markedly decreased but was statistically significant (*F* = 27.85, *p* < 0.001, Figure 3).

### Correlation Between Postoperative 1-Year Thoracic Cavity Volume and Pulmonary Function Test

**FIGURE 4:**
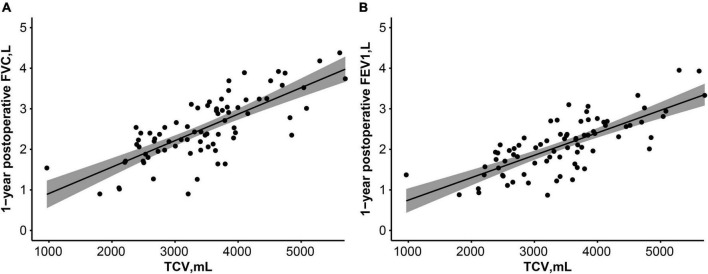
Correlation between postoperative 1-year TCV and pulmonary function test [FVC, L **(A)**, FEV1, L **(B)**]. Lines and dark areas show the regression lines and 95% confidence interval, respectively. FVC, forced vital capacity; FEV1, forced expiratory volume in 1 s.

Postoperative 1-year TCV was closely correlated with forced vital capacity [Pearson’s correlation coefficient = 0.758, 95% confidence interval (CI) 0.664–0.839, *p* < 0.001] and forced expiratory volume in 1 s (Pearson’s correlation coefficient = 0.751, 95% CI, 0.635–0.834, *p* < 0.001, Figure 4).

### Predictive Factors for Thoracic Cavity Volume Change

We constructed a multivariate regression model using clinically relevant variables and lung volume. The restrictive group (vs. the obstructive group), taller recipients, lower recipient BMI, and higher donor pTLC/recipient TCV were found to be independently related to increasing ΔTCV (Table 2).

**TABLE 2:** Multivariable linear regression model for changes in the thoracic cavity volume after lung transplantation.

	B	Standard error	*p*-value
Restrictive group	1,666.000	227.700	<0.001
Age	−0.009	7.333	0.998
Male	−315.500	224.900	0.164
Height, cm	38.220	15.760	0.017
BMI, kg/m^2^	−47.710	22.640	0.038
Donor pTLC/recipient TCV,%	2.804	1.057	0.009
Lung volume reduction	−156.500	170.900	0.362
R^2^ for the model	0.632		

*BMI, body mass index; TCV, thoracic cavity volume; pTLC, predicted total lung capacity.*

## Discussion

**FIGURE 5:**
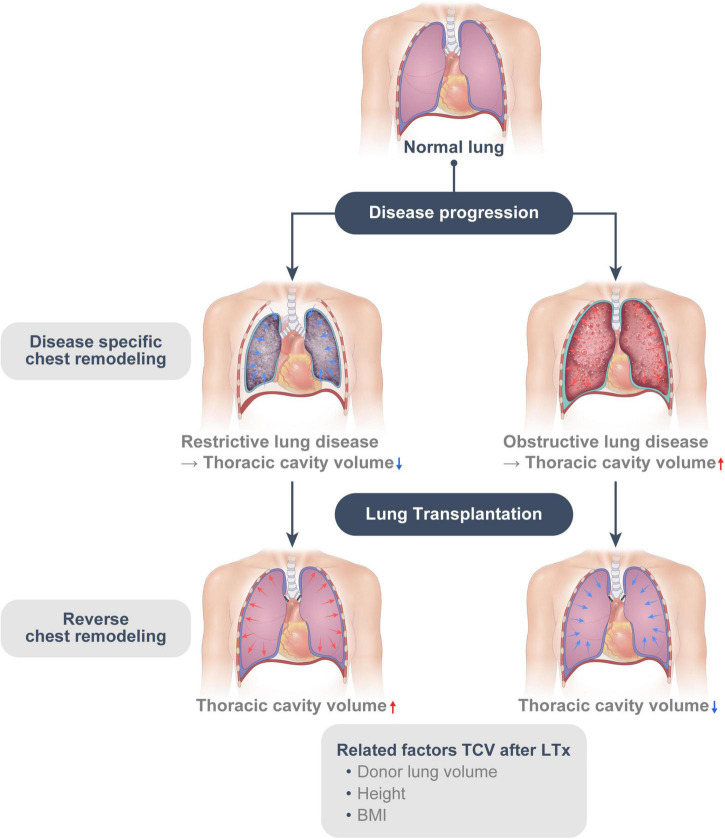
The disease-specific chest remodeling caused by restriction and hyperinflation is at least, in part, reversible. (Reverse chest remodeling).

In this study, we found that the restrictive group had smaller TCV than the obstructive group preoperatively. TCV was found to have increased in the restrictive group and decreased in the obstructive group after LTx. The restrictive group had still smaller TCV than the obstructive group at 1-year post-operation, suggesting that the disease specific chest remodeling—due to restriction and hyperinflation—seems to be partly reversible. In multivariable analysis, disease groups (Restrictive vs. Obstructive), height, BMI, and donor lung size (Donor pTLC/recipient TCV) were associated with the change of TCV (Figure 5).

Due to the unavailability of donor organs, some degree of size mismatching is inevitable in clinical settings; however, there are still controversies about the most suitable size for LTx. Guidelines published in 2003 stated that a donor pTLC between 75% and 125% of the recipient pTLC is considered acceptable and did not cause any adverse clinical or functional effects (14). In this study, we showed that the chest volume changed after LTx and the changes were associated with donor lung size, suggesting that the chest wall can adjust to the newly transplanted lungs. This provides the theoretical background of the wide range of acceptable lung sizes in current guidelines. Post-transplant chest change appeared to proceed in the opposite direction to the preoperative disease-specific chest remodeling. We referred to this phenomenon as “reverse chest remodeling.”

Some studies have implied that disease-specific chest remodeling is reversible after LTx. Wilkens et al. showed reversibility of breathing pattern and chest wall volume during exercise after LTx. They measured breathing patterns and chest wall volumes using opto-electronic plethysmography (OEP) among patients with cystic fibrosis (CF) (*n* = 9), pulmonary fibrosis (PF) (*n* = 9), and COPD (*n* = 21); transplanted patients (*n* = 16); and healthy participants (*n* = 10). Patients with CF, PF, or COPD showed distinct breathing patterns. However, after LTx, the breathing pattern of the transplanted patients became similar to those of healthy participants (9). Tamm et al. analyzed perioperative TLC of 82 patients who underwent heart-lung transplantation (HLTx). They found that the postoperative TLC approached the pTLC of the recipient after HLTx, regardless of the underlying disease, preoperative TLC, or donor pTLC (15). Additionally, Chacon et al. investigated patients who underwent either HLTx (*n* = 6) or bilateral LTx (*n* = 9) in whom the postoperative TLC was significantly correlated with pTLC (*r* = 0.724, *p* = 0.002) but not with preoperative TLC (*r* = 0.148, *p* = not significant) (16).

In this study, the restrictive group still had smaller TCV than the obstructive group at 1 year after LTx, and the difference in postoperative 1-year TCV between the groups still existed after adjustment for pTLC, which suggests the incompletion of the reverse remodeling. To investigate the factors associated with reverse remodeling, we performed a multivariable analysis for the factors affecting ΔTCV. Donor pTLC/recipient TCV was positively correlated with ΔTCV in multivariable analysis and the Pearson’s correlation test (Pearson’s correlation coefficient = 0.537, 95% CI, 0.370–0.670, *p* < 0.001, Supplementary Figure 2). Larger donor pTLC/recipient TCV represents a larger donor lung compared to the diseased thoracic cavity. Therefore, our findings suggest that a constricted thoracic cavity in patients with restrictive lung disease can expand when larger donor lungs are used.

Eberlein et al. investigated 812 bilateral LTx cases from the lung transplant outcome group, a United States National Institutes of Health-sponsored, multicenter, prospective cohort study designed to evaluate risk factors for primary graft dysfunction (PGD). They found that patients with IPF accounted for 43.4% of undersized cohorts (pTLC ratio ≤ 1), and IPF was the most common diagnosis in the undersized cohorts (17). This implies that smaller organs are preferred for patients with restrictive disease because of the risk of complications owing to size mismatch in actual clinical settings. However, in the present study, there was no significant difference in donor/recipient pTLC ratio between the groups. Among patients without lung volume reduction, the restrictive group had significantly lower donor/recipient pTLC ratio compared with that in the obstructive group (98.9 ± 13.9% in restrictive group vs. 109.5 ± 15.7% in obstructive group, Supplementary Table 1). Furthermore, lower lobectomy and multiple resections tended to be performed more frequently in the restrictive group than in the obstructive group. Larger resection of lungs could result in the implantation of smaller lungs, and this might be one of the reasons for smaller TCV in patients with restrictive disease. Some studies have suggested the benefits of an oversized graft, as it delays bronchiolitis obliterans syndrome and has better survival outcomes (18–20). Additionally, Eberlein et al. (1, 17) reported that an oversized graft was not associated with increased postoperative complications. Similarly, we observed a positive correlation between postoperative 1-year TCV and PFT. These suggest an association between the preference of smaller organs in the restrictive group and worse outcomes.

The National Emphysema Treatment Trial (NETT) showed that lung volume reduction (LVR) surgery could improve lung function, exercise capacity, and survival in selected patients with COPD (21, 22). In COPD, the diaphragm is flattened and straightened, which leads to asynchronous movement of the chest wall (23). Zoumot et al. assessed 26 patients who underwent LVR [surgical (*n* = 9) or bronchoscopic (n = 7)] or a sham/unsuccessful bronchoscopic treatment (control subjects, *n* = 10) (24). Patients who underwent LVR showed significant radiologic evidence of decreased lung volume and improved chest wall asynchrony. This study showed that TCV decreased after LTx in the obstructive group, which suggests that COPD patients who undergo LTx might have improved respiratory mechanics.

Height was positively correlated with ΔTCV. Taller patients are likely to have a larger TCV before a lung disease develops and may have the potential to have increased TCV after LTx. In contrast, BMI was negatively correlated with ΔTCV. Obese patients are known to have decreased functional residual capacity (FRC) (25, 26). One study showed that obesity affects the shape of the diaphragm, and the diaphragm was displaced cranially in obese patients (26). Those results are consistent with the result of a separate study showing that BMI was negatively correlated with the height of the diaphragm as measured by posteroanterior chest radiography (27). Such results show that obesity can increase abdominal pressure, displace the diaphragm cranially, and restrict TCV recovery after LTx.

Our study has some limitations. This was a retrospective study conducted in two Korean transplantation centers. The patients included in this study were all Koreans. Lung volumes or TCV varies among races, and pTLC is not specifically calculated for Asians only. Therefore, the donor pTLC could be different from the actual TLC of donors and TCV measured by CT in our study population. In the study period, LVR was performed quite frequently (33.7%). Also, we did not employ the delayed sternum closure strategy that is used for oversized grafts. In case of oversized grafts, graft volume reductions are frequently needed to close the chest. However, some centers recently reported that delayed chest closure may be beneficial in those situations (28, 29). Delayed chest wall closure provides time for the patient to recover from ischemic-reperfusion injury and allows the chest wall to become more compliant. This might make transplantation of larger lungs without graft reduction possible. Nevertheless, further studies are needed to reveal the mechanism underlying the effect of delayed chest wall closure on postoperative TCV and LTx outcomes.

In conclusion, disease-specific chest remodeling caused by restriction and hyperinflation is at least partly reversible. After LTx, the chest remodeling appears to occur in the opposite direction to the disease-specific remodeling caused by the underlying lung disease in recipients. Additionally, this “reverse chest remodeling” might be affected by donor lung size, height, and BMI; furthermore, surgeons prefer smaller organs for restrictive disease and larger organs for obstructive disease; this could restrict “reverse chest remodeling” and be associated with impaired lung function and respiratory mechanics. Therefore, transplant surgeons should consider this remodeling for size matching and donor LVR.

## Data Availability Statement

The data that support the findings of this study are available from the corresponding author upon reasonable request.

## Ethics Statement

This retrospective study was approved by the Institutional Review Board of each hospital (Severance Hospital: 4-2020-0671 and Ajou University Hospital: AJIRB-MED-MDB-20-245). Written informed consent for participation was not required for this study in accordance with the national legislation and the institutional requirements.

## Author Contributions

WY, CP, and SH contributed to conception and design of the study. WY, CP, SY, and JJ organized the database. WY and JS performed the statistical analysis. WY wrote the first draft of the manuscript. CP, HP, JL, SY, JS, JJ, and SH wrote sections of the manuscript. All authors contributed to manuscript revision, read, and approved the submitted version.

## Conflict of Interest

The authors declare that the research was conducted in the absence of any commercial or financial relationships that could be construed as a potential conflict of interest.

## Publisher’s Note

All claims expressed in this article are solely those of the authors and do not necessarily represent those of their affiliated organizations, or those of the publisher, the editors and the reviewers. Any product that may be evaluated in this article, or claim that may be made by its manufacturer, is not guaranteed or endorsed by the publisher.

## Supplementary Material

Supplementary Figure 1 Thoracic cavity volume measurement using a threshold-based three-dimensional segmentation technique. **(A)** A chest CT image of a 56-year-old male patient with IPF before lung transplantation shows subpleural interstitial fibrosis and honeycombing appearances. **(B)** Both lungs are selected using a threshold-based automatic segmentation technique ranging from −200 to −1,024 HU. The selected area is displayed in green. **(C)** To excluded large airways, trachea and main bronchi are carefully selected manually. Selected bilateral main bronchi are shown in pink. **(D)** Anatomical lung volume is measured after careful visual inspection to confirm three-dimensional lung enucleation. The thoracic cavity volume of this patient is 2,890 cm^3^ before lung transplantation. **(E)** After lung transplantation, the thoracic cavity volume was measured in the same way in the chest CT images. A chest CT images 1 year after lung transplantation of this patient shows no evidence of pulmonary fibrosis. **(F)** The thoracic cavity volume measured by chest CT is 3,985 cm^3^.

Supplementary Figure 2 Correlation between ΔTCV and Donor pTLC/recipient TCV (Pearson’s correlation coefficient = 0.537, 95% CI, 0.370–0.670; *p* < 0.001). Lines and dark areas show the regression lines and 95% confidence interval, respectively. TCV, Thoracic cavity volume; pTLC, predicted total lung capacity.

Supplementary Table 1 Patient characteristics with/without volume reduction.
