# Supplementary figures and images for: Changes in Thoracic Cavity Volume After Bilateral Lung Transplantation
Source: Front Med (Lausanne). 2022 May 26;9:881119. doi: 10.3389/fmed.2022.881119 (PMC9204381; doi:10.3389/fmed.2022.881119)

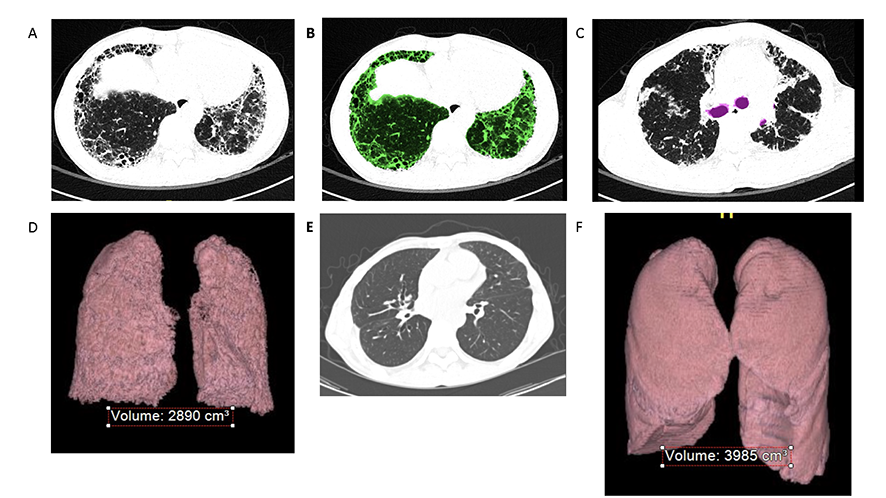

Supplement: Supplementary Figure 1 — Thoracic cavity volume measurement using a threshold-based three-dimensional segmentation technique. (A) A chest CT image of a 56-year-old male patient with IPF before lung transplantation shows subpleural interstitial fibrosis and honeycombing appearances. (B) Both lungs are selected using a threshold-based automatic segmentation technique ranging from −200 to −1,024 HU. The selected area is displayed in green. (C) To excluded large airways, trachea and main bronchi are carefully selected manually. Selected bilateral main bronchi are shown in pink. (D) Anatomical lung volume is measured after careful visual inspection to confirm three-dimensional lung enucleation. The thoracic cavity volume of this patient is 2,890 cm3 before lung transplantation. (E) After lung transplantation, the thoracic cavity volume was measured in the same way in the chest CT images. A chest CT images 1 year after lung transplantation of this patient shows no evidence of pulmonary fibrosis. (F) The thoracic cavity volume measured by chest CT is 3,985 cm3. [file Image_1.TIFF]

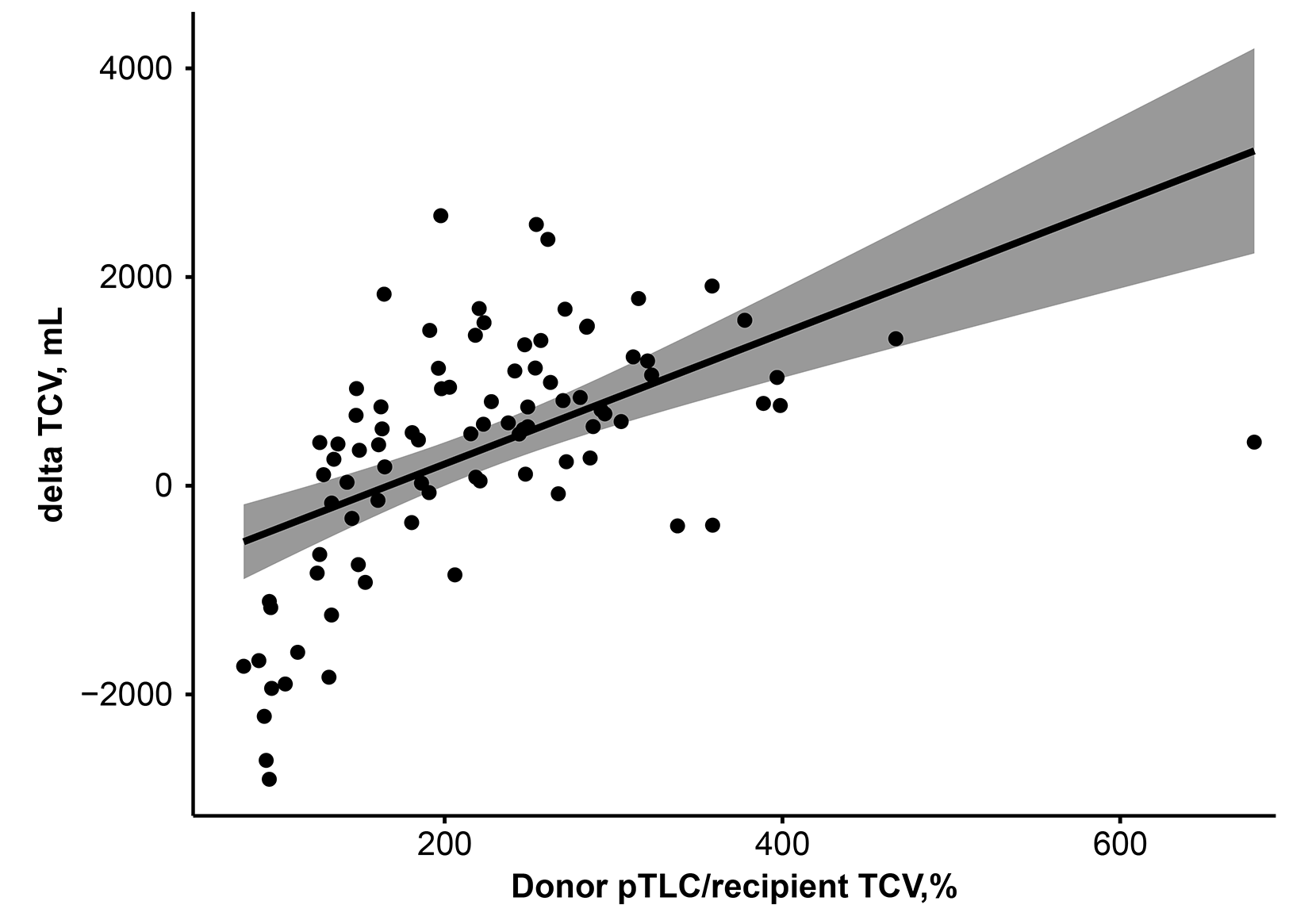

Supplement: Supplementary Figure 2 — Correlation between ΔTCV and Donor pTLC/recipient TCV (Pearson’s correlation coefficient = 0.537, 95% CI, 0.370–0.670; p < 0.001). Lines and dark areas show the regression lines and 95% confidence interval, respectively. TCV, Thoracic cavity volume; pTLC, predicted total lung capacity. [file Image_2.TIF]
