# Supplementary material for: Changes in Thoracic Cavity Volume After Bilateral Lung Transplantation
Source: Front Med (Lausanne). 2022 May 26;9:881119. doi: 10.3389/fmed.2022.881119 (PMC9204381; doi:10.3389/fmed.2022.881119)
Supplement: Supplementary Table 1 — Patient characteristics with/without volume reduction. [file Table_1.DOCX]

Table S1. Patient characteristics with/without volume reduction

|  | **Without lung volume reduction** | | | **With lung volume reduction** | | |
| --- | --- | --- | --- | --- | --- | --- |
|  | **Restrictive**  **(n=41)** | **Obstructive**  **(n=18)** | **p-value** | **Restrictive**  **(n=22)** | **Obstructive**  **(n=8)** | **p-value** |
| **Age, years** | 52.0 ± 9.5 | 43.2 ± 14.1 | 0.022 | 49.4 ± 11.9 | 42.2 ± 16.4 | 0.200 |
| **Male** | 27 (65.9%) | 8 (44.4%) | 0.210 | 11 (50.0%) | 2 (25.0%) | 0.421 |
| **Height, cm** | 166.2 ± 7.2 | 163.3 ± 6.8 | 0.146 | 160.9 ± 6.4 | 163.8 ± 4.7 | 0.264 |
| **BMI, kg/m^2^** | 21.8 ± 3.9 | 17.9 ± 3.0 | <0.001 | 19.9 ± 2.8 | 18.3 ± 5.5 | 0.454 |
| **Diagnosis, n (%)** |  |  | <0.001 |  |  | 0.006 |
| **Bronchiectasis** | 1 (2.4%) | 4 (22.2%) |  | 2 (9.1%) | 1 (12.5%) |  |
| **COPD** | 0 (0.0%) | 2 (11.1%) |  | 0 (0.0%) | 1 (12.5%) |  |
| **CTD-ILD** | 6 (14.6%) | 0 (0.0%) |  | 4 (18.2%) | 0 (0.0%) |  |
| **GVHD** | 2 (4.9%) | 4 (22.2%) |  | 2 (9.1%) | 3 (37.5%) |  |
| **IPF** | 30 (73.2%) | 1 (5.6%) |  | 13 (59.1%) | 0 (0.0%) |  |
| **LAM** | 0 (0.0%) | 5 (27.8%) |  | 0 (0.0%) | 2 (25.0%) |  |
| **Other – ILD** | 2 (4.9%) | 2 (11.1%) |  | 1 (4.5%) | 1 (12.5%) |  |
| **FEV1 % predicted** | 46.2 ± 15.5 | 24.4 ± 14.0 | <0.001 | 41.3 ± 15.9 | 26.5 ± 11.3 | 0.023 |
| **FVC % predicted** | 38.6 ± 12.0 | 47.8 ± 18.1 | 0.059 | 35.2 ± 12.0 | 47.8 ± 9.9 | 0.013 |
| **FEV1/FVC (%)** | 88.4 ± 6.7 | 33.9 ± 12.4 | <0.001 | 88.9 ± 9.4 | 43.5 ± 15.3 | <0.001 |
| **Lung volume reduction** | 0 (0.0%) | 0 (0%) |  | 22 (100.0%) | 8 (100.0%) |  |
| **Procedure for lung volume reduction** |  |  |  |  |  | 0.297 |
| **Lower lobectomy** | 0 (0.0%) | 0 (0.0%) |  | 2 (9.1%) | 0 (0.0%) |  |
| **Middle lobectomy** | 0 (0.0%) | 0 (0.0%) |  | 0 (0.0%) | 1 (12.5%) |  |
| **Middle lobectomy + Wedge** | 0 (0.0%) | 0 (0.0%) |  | 2 (9.1%) | 0 (0.0%) |  |
| **Wedge, multiple** | 0 (0.0%) | 0 (0.0%) |  | 12 (54.5%) | 3 (37.5%) |  |
| **Wedge, single** | 0 (0.0%) | 0 (0.0%) |  | 6 (27.3%) | 4 (50.0%) |  |
| **Donor/recipient size discrepancy** |  |  |  |  |  |  |
| **Recipient pTLC, mL** | 5552.3 ± 787.8 | 5285.6 ± 793.8 | 0.237 | 5051.0 ± 758.4 | 5196.6 ± 415.8 | 0.613 |
| **Donor pTLC, mL** | 5437.7 ± 797.5 | 5758.2 ± 1018.7 | 0.198 | 5711.7 ± 788.8 | 5921.5 ± 687.3 | 0.512 |
| **Donor pTLC/recipient pTLC, %** | 98.9 ± 13.9 | 109.5 ± 15.7 | 0.012 | 114.2 ± 15.2 | 114.3 ± 14.3 | 0.989 |
| **Donor pTLC/recipient TCV, %** | 249.2 ± 97.8 | 124.9 ± 35.8 | <0.001 | 273.9 ± 72.8 | 152.3 ± 49.5 | <0.001 |
| **Recipient TCV** |  |  |  |  |  |  |
| **Preop TCV, mL** | 2417.9 ± 737.6 | 4885.3 ± 1322.9 | <0.001 | 2217.1 ± 649.9 | 4162.6 ± 1156.7 | 0.002 |
| **1-Year TCV, mL** | 3393.5 ± 951.4 | 3836.2 ± 890.7 | 0.099 | 2909.4 ± 780.7 | 3429.6 ± 884.4 | 0.130 |
| **ΔTCV, mL** | 975.6 ± 738.9 | -1049.1 ± 1000.1 | <0.001 | 692.2 ± 478.6 | -733.0 ± 951.5 | 0.003 |
| **1-Year PFT** ^a^ |  |  |  |  |  |  |
| **FVC % predicted** | 64.0 ± 18.6 | 69.2 ± 15.3 | 0.344 | 66.5 ± 22.2 | 69.0 ± 14.1 | 0.810 |
| **FEV1 % predicted** | 72.8 ± 21.6 | 79.5 ± 19.4 | 0.302 | 71.0 ± 22.7 | 67.4 ± 7.2 | 0.556 |
| **FEV1/FVC (%)** | 85.3 ± 11.0 | 89.5 ± 11.4 | 0.229 | 81.8 ± 11.1 | 80.6 ± 7.6 | 0.829 |
